# Aerobic bacterial flora of biotic and abiotic compartments of a hyperendemic Zoonotic Cutaneous Leishmaniasis (ZCL) focus

**DOI:** 10.1186/s13071-014-0517-3

**Published:** 2015-01-29

**Authors:** Naseh Maleki-Ravasan, Mohammad Ali Oshaghi, Davoud Afshar, Mohammad Hossein Arandian, Sara Hajikhani, Amir Ahmad Akhavan, Bagher Yakhchali, Mohammad Hasan Shirazi, Yavar Rassi, Reza Jafari, Koorosh Aminian, Reza Ali Fazeli-Varzaneh, Ravi Durvasula

**Affiliations:** Department of Medical Entomology and Vector Control, School of Public Health, Tehran University of Medical Sciences (TUMS), Tehran, Iran; Malaria and Vector Research Group (MVRG), Biotechnology Research Center (BRC), Pasteur Institute of Iran, Tehran, Iran; Department of Pathobiology, School of Public Health, Tehran University of Medical Sciences (TUMS), Tehran, Iran; Isfahan Health Research Station, National Institute of Health Research (NIHR-IHRS), Esfahan, Iran; Department Industrial and of Environmental Biotechnology, National Institute of Genetic Engineering and Biotechnology, (NIGEB), Tehran, Iran; Isfahan Province Health Center No1, Isfahan University of Medical Sciences, Isfahan, Iran; Varzaneh Health Center, Isfahan University of Medical Sciences, Isfahan, Iran; Department of Internal Medicine, University of New Mexico, Albuquerque, New Mexico

**Keywords:** *Phlebotomus papatasi*, Microflora, Paratransgenesis, Zoonotic Cutaneous Leishmaniasis, Iran

## Abstract

**Background:**

Identification of the microflora of the sand fly gut and the environmental distribution of these bacteria are important components for paratransgenic control of *Leishmania* transmission by sand flies.

**Methods:**

Biotic and abiotic bacterial communities of four compartments of a hyper-endemic focus of Zoonotic Cutaneous Leishmaniasis (ZCL) were investigated using 16S ribosomal DNA sequencing and phylogenetic tree construction. These compartments include *Phlebotomus papatasi’s* gut, skin and intestinal tract of great gerbil *Rhombomys opimus*, the gerbil nest supplies, and plant food sources of the vectors and reservoirs.

**Results:**

Sequence homology analysis using nine available 16S rDNA data bases revealed 40, 24, 15 and 14 aerobic bacterial species from the vector guts, the gerbil bodies, the gerbil nests, and the plants, respectively. The isolated bacteria belong to wide ranges including aerobic to facultative anaerobic, pathogen to commensals, sand fly oviposition inducers, land to air and ocean habitats, animal and human probiotics, and plant growth-promoting rhizobacteria. Matching data analysis suggested that the adult *P. papatasi* gut bacteria could be acquired from three routes, adult sugar feeding on the plant saps, adult blood feeding on the animal host, and larval feeding from nest supplies. However, our laboratory experiment showed that none of the bacteria of the reservoir skin was transmitted to female sand fly guts via blood feeding. The microflora of sand fly guts were associated with the sand fly environment in which the predominant bacteria were *Microbacterium, Pseudomonas*, and *Staphylococcus* in human dwellings, cattle farms, and rodent colonies, respectively. *Staphylococcus aureus* was the most common bacterium in sand fly guts. Presence of some sand fly ovipoisition inducers such *Bacillus* spp. and *Staphylococcus saprophyticus* support association between gut flora and oviposition induction.

**Conclusions:**

Results of this study showed that *Bacillus subtilis* and *Enterobacter cloacae* particularly subsp. *dissolvens* are circulated among the sand fly guts, the plants, and the sand fly larval breeding places and hence are possible candidates for a paratransgenic approach to reduce *Leishmania* transmission*.*

## Background

Leishmaniases are worldwide distributing sand fly-borne parasitic diseases with 1.4 million new cases and 20–30 thousand deaths annually. Due to complexity of life cycle of *Leishmania* spp. multifaceted intervention strategies are needed to prevent and control of the disease [1,2]. From the leishmaniasis spectrum, Zoonotic Cutaneous Leishmaniasis (ZCL), a neglected tropical disease, is a public health problem with a clear and disturbing increase in the number of cases in some areas of the world [3,4]. *Leishmania major* is widely distributed in various populations of rodents in arid and savannah regions [4,5] and transmitted by the Afro-Asian vector of ZCL, *Phlebotomus papatasi* Scopoli 1786, one of the most prevalent species among the *Phlebotomus* genus in indoor and outdoor places [6-10].

The disease is endemic in many rural districts of Iran, in 17 out of 31 provinces [11], so that it is still a great health problem and of research interest to many investigators. Rodents belonging to the subfamily Gerbillinae are the main reservoir hosts of ZCL in Iran and other countries where ZCL is endemic [12,13]. In general, gerbils are the most abundant mammals reported from natural ecosystems of Old World deserts [14].

The great gerbil, *Rhombomys opimus* (Cricetidae: Gerbillinae), is widely distributed in arid and semi arid habitats, mostly in sandy or clay desert areas throughout Central Asia, including Northwestern China, Mongolia, Russia, Kazakhstan, Iran, Afghanistan and western Pakistan [15-17]. In Iran it is widely distributed in central and northeast parts of the country [16,18,19]. Based on mitochondorial DNA cytochrome B (cytB) gene, at least two subspecies *R. opimus sodalis* and *R. opimus sargadensis* have been reported in Iran [20]. Because their burrows are a long-standing and important feature of the landscape, many other animal species such as Phlebtominae sand flies use them for shelter. Three coexisting *Leishmania* parasites, *L. major*, *L. turanica*, *L. gerbilli* and the bacterium *Yersinia pestis* have been isolated from this rodent and its corresponding insect vectors, which indicate that the great gerbil is a major reservoir that can maintain natural infection of leishmaniasis or plague [5,10,18,21,22]. The rate of infection of *R. opimus* by *L. major* is normally high and may vary from 55.8% to 92.5% in endemic areas [5,18]. The parasite can persist in the great gerbils for up to 25 months [23].

The primary diet of great gerbils is herbivorous (Folivore, Frugivore, and Granivore) and they cache these foods in complex tunnel systems. Living in desert habitats, this gerbil must rely on metabolic water found in succulent plants of family Chenopodiaceae (*Climacoptera* spp., *Salsola* spp., *Suaeda* spp.) [24,25]. Although their diet may vary according to the changes of plant species and coincides with the variations in the plants’ phenology [26], in central Iran, gerbils are commonly folivorous on *Haloxylon* spp. and *Salsola* spp. These plants constitute the main source of gerbil food because they have higher levels of water and mineral salts compared with other plants [27].

The insect alimentary canal is the main colonizing site of many microorganisms. Sand flies acquire bacteria at the larval stage from food and the breeding soil, and at the adult stage through contaminated sugar meals derived from plant leaves and fruits or aphid honeydew [28]. Sand fly females may also ingest bacteria while feeding on a blood meal; however, blood meals are usually sterile, while sugar meals from different sources may contain a variety of contaminating microorganisms [29]. These microbes are involved in many aspects of the host life including nutrition, reproduction, tolerance to environmental perturbations, maintenance and/or enhancement of host immune system homeostasis, defense, speciation, mucosal barrier fortification, xenobiotic metabolism, and pathogen transmission ability [29-35]. Among these, the role of midgut-associated bacteria in food digestion has been demonstrated in several insect species [34]. These indigenous bacteria are essential sources of carbohydrates improving digestion efficiency of plant-derived polymers such as lignin, hemicellulose and cellulose, xylan and pectin [36] and may also contribute to lipid and protein digestion [37].

Female sand flies need blood for egg production, but sugar is their main source of energy and the only food taken by males [38]. The sugar feeding behavior of sand flies, therefore, influences longevity and fecundity, dispersal, host seeking behavior and ultimately blood feeding and disease transmission [39-42]. According to the literature, sand flies obtain sugar meals mainly from honeydew excreted by aphids and coccids [43,44] and by feeding directly on tissues of plants in the field [45,46].

Generally, vector control is an effective and the simplest method to control vast majority of vector-borne diseases [47]. However in recent years, application of pesticides have been problematic because of their environmental toxicity, adverse effects on human health and the emergence of insecticide resistance in many countries [48].

Paratransgenesis is a Trojan-horse approach in which symbiotic bacteria, fungi, or viruses of the vector insect are genetically manipulated to deliver effector proteins that block development or transmission of the pathogen (vector competence). This approach attempts to decrease pathogen transmission without adverse effects on vectors themselves. Further, it is considered as a gene delivery mechanism to the host and indigenous bacterial flora of the host vector [34]. Bacterial symbionts of blood sucking bugs [49], tsetse flies [50], mosquitoes [51-55], American cockroach [56] and sand flies [57,58], as well as symbiotic viruses of *An. gambiae* [59] and *Aedes aegypti* [60], have been identified and in some cases successfully used to reduce or eliminate carriage of pathogens by host arthropods.

Multitrophic interactions are now recognized as being very important in understanding the complexity of the natural world. For example, during phytophagy or haematophagy, insects encounter microbiota on the surface of the host and their released metabolic products; likewise, the host is also exposed to microbial products released from both sides of the insect alimentary canal [34]. The role of microbiota as a fourth partner in behavioral aspects of vector-parasite-host interactions has been neglected for long time. Information gained from the study of these interactions can form the interface between biological control and restoration, which should be considered as part of biological control.

In this study, the presence and distribution of gut microbiota of male and female *P. papatasi*, the main vector of ZCL, were investigated from the following sources in the hyper-endemic focus of ZCL, Isfahan province, Iran: the exposed areas of skin surface, faeces and viscera of the great gerbil, *R. opimus,* the animal’s nest materials which include soil, vegetarian food residues, and two plants of *Hyloxylon* sp. and *Salsola* sp. as the food sources of both vector and reservoir. The results of this study may lead to identification of an appropriate bacterial candidate for genetic manipulation and delivery of effector molecules to diminish leishmaniasis transmission, using a paratransgenic strategy.

## Methods

### Study area

**Figure 1:**
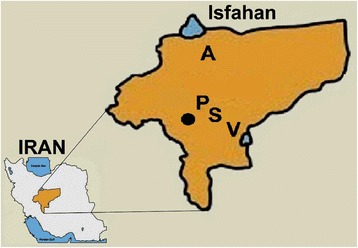
**Map of study area in the hyperendemic ZCL focus of Isfahan province, central of Iran.** Locations are, A: Abbas Abad (Badrood), P: Parvaneh (Habibabad), S: Sejzi and V: Varzaneh. Solid dot: Isfahan city.

The study was conducted in five locations of four districts of the Isfahan province, a well-known hyper endemic ZCL focus in central Iran (Figure 1). Biological and non-biological samples were collected from different biotypes including excavated rodent colonies, semi-desolated cattle farm, uninhabited home, and deserts in the vicinity of villages and cities of the district.

### Sample collection

#### Sand fly collection

Initially funnel traps and sticky traps were used to evaluate the sand fly populations in the region. In order to study the sand fly gut mirobiota, live sand flies were collected using different methods including CDC light traps, car traps, and aspirator during the months of June and July 2012. The traps were set adjacent to rodent burrows and animal shelters between the hours of 20:00 PM to 4:00 AM next day. Car traps involved a just parked vehicle used to attract sand flies in the vicinity of rodent burrows at nighttime. By this method the sand flies landing on the car were collected using a mouth aspirator and battery operated torch. Mouth aspirator and battery operated torch also were used to collect sand flies from bathrooms and toilets within human dwellings. Sand flies were transferred alive to the National Institute of Health Research, Isfahan Health Research Station (NIHR-IHRS) laboratory. The specimens were divided into two groups: 1) sand flies were immediately microdissected and transferred into brain heart infusion (BHI) broth culture medium, and 2) sand flies were offered blood meals on their natural host, *R. opimus,* that were reared in the animal unit of the center. The guts of the second group were microdissected and cultured in the medium 24 hours after blood meal ingestion. The specimens were identified after microdissection and only *P. papatasi* specimens were tested for gut microbiota.

#### Rodent collection

Active colonies of gerbils were identified and animal collections were performed in summer season using a Sherman live-trap (30 cm × 15 cm × 15 cm wire mesh) baited with a sliced cucumber. Approximately 15–20 live traps per day were used in each location. The traps were set close to a burrow entrance 2–3 h before the initiation of diurnal activity in the morning and in the evening when the temperature was not very hot. The traps were checked at noon and in the late evening before sunset. Collected rodents were fed sliced carrots until microbiological studies.

#### The nest materials

To examine acquirement, association, and probable circulation of bacteria among the sand fly gut, rodent skin, and food sources within the rodent nest, the soil, food supplies, and wool fibres within the nest were collected from the same colony where sand flies and rodents were trapped. For this purpose the rodent burrows that were built on clay hills were selected for excavation. Sampling was performed from different parts of the nest for bacterial investigation.

#### Plants: *hyloxylon* sp. and *salsola* sp

Sampling of the plants was confined to the available plants of *Haloxylon* sp. and *Salsola* sp., the two prevalent plants in the central desert of Iran. The whole plant of *Salsola* sp. (bushes) and the leaves and green branches of *Haloxylon* sp. were cut and placed in a sterile clean plastic bag and labeled with the given rodent colony.

### Isolation of bacteria

#### Sand fly guts

Isolation of sand fly guts was conducted in a sterile environment under a microbiological lab hood on a sterile glass slide. Before dissection, individual flies were surface sterilized for 2 min in 70% ethanol. The gut from each sand fly was micro-dissected and homogenized in test tubes with screw tops containing 5 cc brain heart infusion (BHI) broth medium. The rest of the dissected insect body was mounted on a slide for morphological identification.

#### The nest materials

The nest materials comprising the plant pieces, wool fibres, and soil samples were collected and transferred to the lab. Plant pieces and wool fibres were gathered in sterile bags and then were immersed in the BHI broth medium. For the soil samples, 0.7 ml of soil sample was collected using 0.7 ml micro tubes and then homogenized in the same medium.

#### The rodent body

The live field captured rhomboids and the laboratory reared *R. opimus* in the NIHR-IHRS animal unit, were anesthetized using intramuscular ketamine hydrochloride (60 mg/kg) and xylazine (5 mg/kg). Sterile cotton swabs (presented in a sterile sealed test tube) were used to swab the exposed area around the auricle, eyelid, muzzle or footpads of the animal, where sand flies choose to take blood meals. The swabs were then placed in BHI broth. Single rodent faeces were collected from the colonies in 0.7 ml micro tubes and then homogenized in the BHI medium. Also a rodent was dissected for swabbing throughout its alimentary canal. All experiments on the rodents were performed in accordance with the guidelines of the Ethical Board of Tehran University of Medical Sciences, Iran.

To test whether the rodent skin bacteria can enter/infect the sand fly gut via blood feeding, a group of unfed female sand flies were allowed to feed on laboratory *R. opimus* specimens mentioned above. The gut microflora of the fed sand flies were tested 24–48 hours post-blood meal as this coincides with the highest growth rate of bacteria as suggested in the literature [30,35].

#### Plants: *Hyloxylon* sp. and *Salsoa* sp.

To examine the surface bacterial flora of the diet plants (Epiphytes), portions of collected samples, were placed into the screw top tubes containing liquid medium. For isolation of the potential bacteria present in the tissue and sap (Endophytes) of the given plants, first about 10 grams of the these plants were surface sterilized with 70% alcohol for two minutes and then their juice was extracted by mortar. Finally the plant juice was poured directly into the BHI broth culture medium.

### Bacteriological methods

#### Culture media

In this research we initially used BHI broth medium. It is a versatile liquid infusion medium, and was chosen as an enriched non-selective medium to promote growth of a diverse range of microbes including nutritionally fastidious and nonfastidious bacteria, aerobic and facultative anaerobic bacteria, from a variety of clinical and nonclinical materials. The transparent test tubes were incubated aerobically at 37°C overnight. After 24–48 hours, opaque test tubes considered as positive were sub cultured in BHI agar medium overnight at the same condition. A test tube containing BHI broth opened near the dissection area constituted our sterility control during the dissection process.

#### Purification of bacterial isolates

To obtain individual pure colonies, the grown bacteria were serially diluted or streaked on agar plates. After 18–24 hours incubation at 37°C area well-isolated discrete colonies were seen. Colonies with different phenotype and morphology were isolated and sub-cultured successively. Pure isolates were partially preserved and partially used for further identification procedures like Gram staining and molecular studies.

### Molecular identification

#### 16S rRNA gene amplification

The purified bacterial colonies isolated from different specimens were tested using sequence analysis of the 16S rRNA gene for molecular identification after initial classical phenotyping and biochemical identifications. Each purified colony was subjected to genomic DNA extraction using either a boiling method (STET buffer) and/or routine phenol/chloroform DNA extraction method for the isolates with hard cell walls that had not yielded proper DNA by the boiling method. The 16S rRNA universal primers 16suF: 5′-GAGTTTGATCCTGGCTCAG-3′ and 16suR: 5′-GTTACCTTGTTACGACTT-3′ [61] were used to amplify a 1.5 kilo base (kb) partial sequence of the 16S rRNA gene. The PCR amplification was performed using Maxime PCR PreMix Kit (i-*Taq*) Cat. No. 25026 in 20 μl reaction mixtures containing 1 μl of 10 μM both forward and reverse primers and 1–2 μl (~0.1 μg) of extracted genomic DNA. Double-distilled water and BHI agar medium were used as DNA extraction and PCR negative controls. The PCR conditions were set as an initial denaturation at 94°C for 10 min, followed by 35 cycles of denaturation at 95°C for 30 s, annealing at 57.5°C for 40 s, and extension at 72°C for 30 s, followed by a final extension at 72°C for 8 min. PCR products were visualized on a 1% agarose gel containing ethidium bromide and using an UV transilluminator.

#### 16S rRNA gene sequencing and analysing

All successfully amplified 16S rRNA amplicons were bidirectionally sequenced via the same amplification primers by Bioneer Company (S. Korea). The probable chimeric sequences were checked with Mallard program [62] for all acquired sequences and the specimens with suspicious sequences removed from data. The consensus of confident sequences was analyzed using nine databases available for 16S rRNA genes of prokaryotes including Greengenes [63], EzTaxon-e [64], NCBI (16S rRNA sequences) [65], NCBI (Nucleotide collection) [66], EMBL [67], DDBJ [68], leBIBI [69], RDP [70] and Blast2Tree [71]. Sequence homology analysis was based on the number and quality of nucleotides in a given sequence and hence appropriate settings and defaults such as cultivable and or non-cultivable, type specimens and or non-type specimens were selected. In case of discrepancies among different databases, species identifications were based on either the most common nomenclature among the results of the nine databases or on the basis of the highest percentage similarity.

The MEGA5 software was used for phylogenetic analyses and tree construction. Position verifications were done using distance (neighbor joining) and parsimony (1000 bootstrap replicates) analyses. The sequences were deposited in GenBank database.

The DNA gyrB PCR method as described by Wang et al. [72], followed by RFLP using suitable restriction enzyme/s were used for identification of the isolates for which 16S rRNA sequences represented more than a single species such as *Shigella flexneri*/*Escherichia coli*, *Stenotrophomonas maltophilia*/*Pseudomonas geniculata*, closely related *Bacillus* species that share a similar genetic background but occupy different ecological niches (*B. thurengiensis, B. anthracis* and *B. cereus*), and subspecies of *Bacillus subtilis*. Those bacteria for which 16S rRNA sequences were identical were normally determined by the EzTaxon database.

#### Contamination controls

In order to verify the findings, bacterial contamination of other parts of the dissected sand flies (except for gut), rodent skin used for sand fly blood feeding, rodent viscera, and environmental bacterial contamination of culture media were examined.

## Results

### Collected samples

In total, 476 biotic and abiotic specimens of the ZCL compartments originated from five locations of Isfahan province were collected and their microflora were examined. They included 390 sand fly guts, 28 rodent skins, 11 rodent feces, 11 rodent nest soils, 12 plant pieces collected within the rodent nest, 2 wool fibre samples, 14 *Haloxylon* sp. samples and 8 *Salsola* sp. samples. Details of the collected samples are given in Table 1.

**Table 1:** **Details and number of specimens used for microbiota analysis**

**Specimen**	**Location**					
	**Varzaneh city**	**Abbas-abad village, Badrood city**		**Parvaneh village, Habib-Abad city**	**Sejzi city**	**Total**
		**Rodent colony**	**Cattle farm**			
Male sandfly	---	7	6	28	---	41
Female sandfly	---	170	92	87	---	349
*R. opimus*	16	3	---	---	8*	27
*Meriones libycus*	1	---	---	---	---	1
Rodent feces	8	3	---	---	---	11
Nest soil	8	3	---	---	---	11
Nest plant pieces	8	4	---	---	---	12
Nest wool fibers	2	---	---	---	---	2
Epiphyte of *Hyloxylon*	4	3	---	---	---	7
Endophyte of *Hyloxylon*	4	3	---	---	---	7
Epiphyte of *Salsola*	1	3	---	---	---	4
Endophyte of *Salsola*	1	3	---	---	---	4
Total	53	202	98	115	8	476

*The origin of R. opimus reared in the Isfahan Health Research Station animal house was Sejzi city.

### Identification of isolated bacteria

Initially, all isolates were identified according to their morphological characteristics. On the basis of the cell morphology (Gram staining) the isolates fell into two main categories of Gram-negative (n = 24) and Gram-positive (n = 45) bacteria. On the basis of the colony morphology (form, elevation, margin, surface, opacity, and chromogenesis) a large variation of bacterial isolates was described. Finally, sequence analysis of 16S rRNA gene revealed 12 isolates from male sand fly guts, 162 isolates from female sand fly guts, 47 isolates from the internal and external parts of rodent bodies, 31 isolates from rodent nest materials, 14 isolates from *Haloxylon* sp. and 7 isolates from *Salsola* sp. plants.

**Figure 2:**
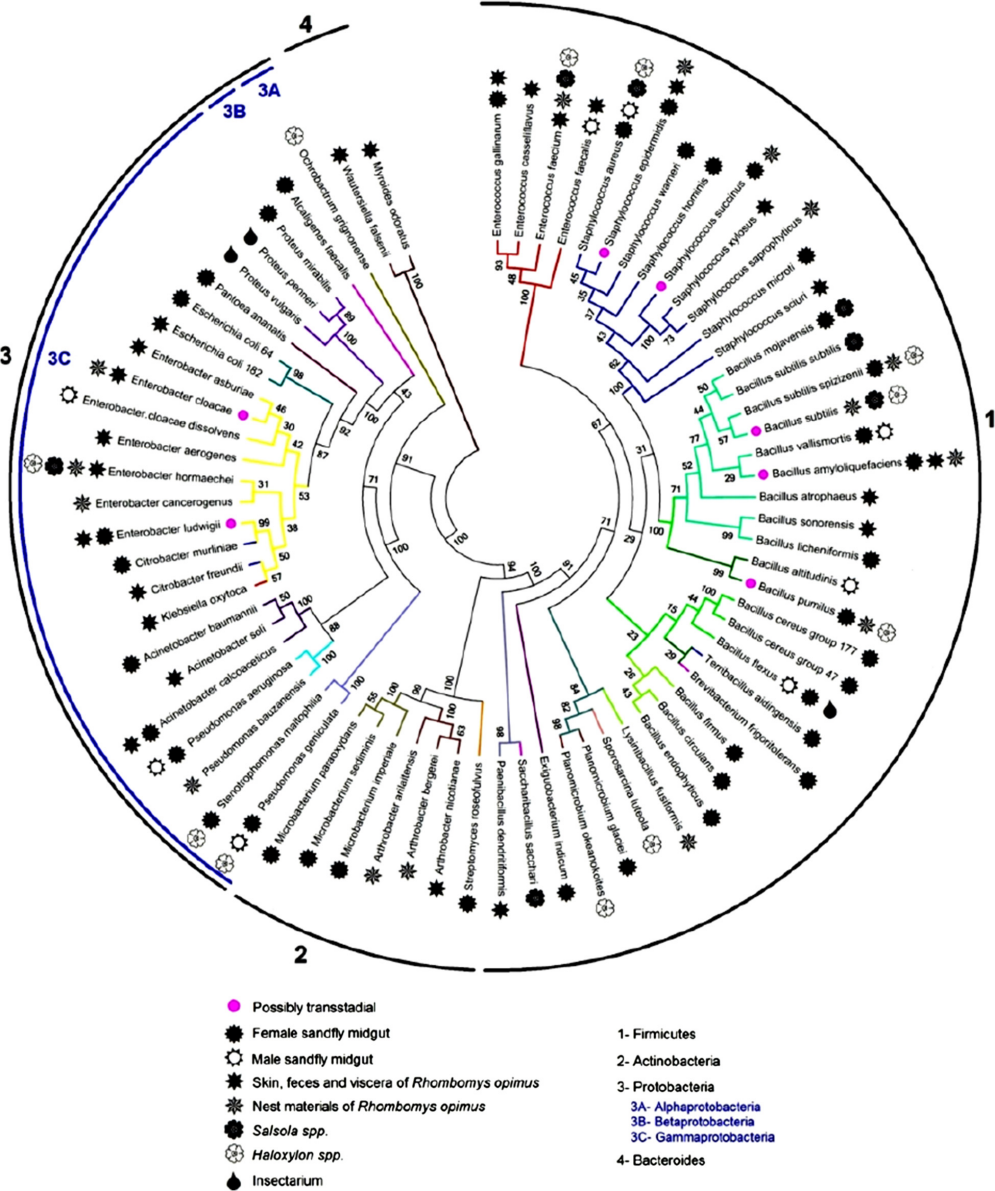
**Phylogenetic relationship of the bacteria isolated from the partners of the ZCL Hyper-endemic focus of Isfahan province, Iran.** Common and similar colors point out groups, complexes and close relatives. Solid and hollow signs represent the source of isolation and numbers around circles indicate bacterial phyla. The tree is drawn to scale, with branch lengths in the same units as those of the evolutionary distances used to infer the phylogenetic tree. Numbers at the nodes indicate percent bootstrap values (1000 replicates).

**Figure 3:**
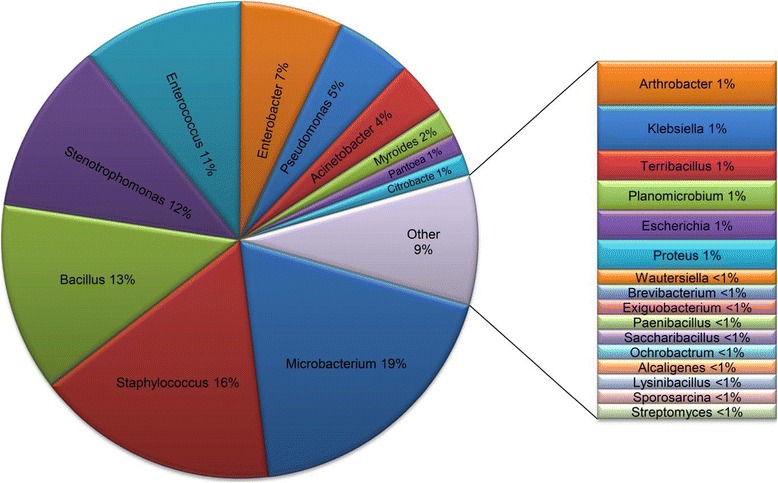
**Categorization and relative abundance of the identified isolated bacteria from the partners of the ZCL Hyper-endemic focus of Isfahan province, Iran.** *Pseudomonas geniculata* isolates are grouped with Genus *Stenotrophomonas*.

In total 273 16 rRNA PCR products were sequenced and the consensus data were deposited in GenBank. [GenBank: JX661713-JX661718 and GenBank: KF254489-KF254756] (Table 2). Molecular identification was performed according to the 16S rRNA gene sequence similarity rates between the amplified specimens and the available data in the nine data banks (Table 2). Molecular identification revealed presence of 40, 24, 15, and 14 bacterial species from vector midgut, reservoir host body, rodent nest supplies, and the vegetarian diet sources, respectively. Phylogenetic relationships of the bacteria species are shown in a diagrammatic representation in Figure 2. They belonged to 4 phyla, 16 families, and 28 genera of bacteria (Table 2). The relative abundance of the bacteria genera is shown in Figure 3. Herein we report 69 bacterial species from four phyla comprising 44% Firmicutes, 34% Proteobacteria, 20% Actinobacteria and 2% Bacteroidetes from the four main components of the hyper-endemic ZCL focus. From 476 biotic and abiotic specimens that were investigated, most specimens contained culturable bacteria; some had two or more species, but in sand fly vectors, 75% of females and 68% of males were gnotobiotic while four plant specimens and one wool fibre of nest material were sterile.

**Table 2:** **Details of the isolated bacteria from biotic and abiotic compartments of Isfahan ZCL focus based on 16S rRNA sequences**

**Classification (Family)**	Isolation source*	AN of the closest relative according to consensus of 9 data bases and BLAST servers** (T = Type strain)	**Reported sources based on the closest relative and GenBank searching**	**Name of the closest relative according to consensus of 9 data bases and BLAST servers**	**The highest similarity score %**	**Genbank AN**
Micrococcocaceae	8	[EzTaxon-e: AJ609630]	cheese; rhizosphere of wheat, tomato; endophyte of wheat, rice; air; soil; rabbit stool; mushroom compost; subsurface water from the China Sea	*Arthrobacter bergerei*	98.30%	[KF254744]
	6	[EzTaxon-e: FQ311875]		*Arthrobacter arilaitensis*	99.22%	[KF254745]
	3	[EzTaxon-e: X80739]	rhizosphere of wheat, tobacco; soil; air; lake; cold desert; dust; vermicompost; as plant growth-promoting rhizobacteria (PGPR)	*Arthrobacter nicotianae*	99.22%	[KF254746]
Microbacteriaceae	2	[EzTaxon-e: HQ219727] (T)	deep-sea sediment	*Microbacterium sediminis*	98.39%	[KF254679]
	2	[EzTaxon-e: X77442]	vermicompost; endophyte of tobacco; air, plant roots	*Microbacterium imperiale*	99.93%	[KF254682]
	2	[Greengenes: EU714342]	human clinical specimens; bovine rumen; raw milk; industrial effluent, rhizoplane of wheat, eucalyptus	*Microbacterium paraoxydans*	100%	[KF254680-81,683-729]
Streptomycetaceae	2	[EzTaxon-e: AB184327] (T)	rhizosphere of sugar beet, wheat, corn, soybean; soil	*Streptomyces roseofulvus*	100%	[KF254735]
Flavobacteriaceae	3,4	[EzTaxon-e: CM001437]	habitat-specific organisms; wet environments; bats; vermicompost; coastal water	*Myroides odoratus*	99.50%	[KF254739-43]
	3	[Greengenes: FM162560]	soil; polluted sediment; clinical samples	*Wautersiella falsenii*	99.67%	[KF254733]
Bacillaceae	2	[EzTaxon-e: AM747813] (T)	arid soil; rhizosphere of elymus, carrot, maize, saffron, tea; endophyte of soybean	*Brevibacterium frigoritolerans*	100%	[KF254755]
	2	[EzTaxon-e: AF295302]	endophyte of cotton, wheat; PGPR; salt lake; sediment; soil; coast; desert; root of glycine max, grapevine; *Bemisia tabaci* honeydew	*Bacillus endophyticus*	99.65%	[KF254667]
	1,2	[EzTaxon-e: AB021185]	maize; seaweed, coral; mine; freshwater pond; fish gut; poultry waste; vermicompost	*Bacillus flexus*	100%	[KF254668-69]
	2	[Greengenes: EF032672]	PGPR; saltmarsh sediment; Marine black sponge	*Bacillus firmus*	100%	[KF254670]
	2	[EzTaxon-e: AY724690] (T)	milk powder; soil; hot spring water; as probiotic bacterium in aquaculture	*Bacillus circulans*	100%	[KF254671-72]
	2,6,9	[Greengenes: FJ549019]	slaughterhouse waste; extremophile; a PGPR with antibacterial and antifungal activity; oil; soil; roots; milk powder	*Bacillus pumilus*	99.74%	[KF254673-76]
	1	[EzTaxon-e: AJ831842] (T)	upper atmosphere; spring soil; rhizosphere of wheat,rice, nut, tobacco	*Bacillus altitudinis*	100%	[KF254585]
	2,3,8	[Greengenes: GU568185]	larvicidal activity against culicidae; biocontrol activity against plant pathogens; great industrial application for production of enzymes, antibiotics, fermented foods and vitamins; Human, veterinary and aquaculture; probiotic; fermented soybean; *Dioscorea zingiberensis*; soil; hami-melon juice, slaughterhouse soil	*Bacillus amyloliquefaciens*	100%	[KF254566,71,77, JX661713]
	2,6,7,9	[leBIBI: AB325584] (T)		*Bacillus subtilis* subsp *spizizenii*	100%	[KF254562,64,65,67,69]
	11	[NCBI,16S: NR_027552] (T)		*Bacillus subtilis* subsp *subtilis*	99%	[KF254580]
	8,9,11	[Greengenes: HM210636]		*Bacillus subtilis*	99.77%	[KF254563,68,70,82]
	2	[NCBI,NC: HE993550]		*Bacillus licheniformis*	100%	[KF254572]
	1,2	[NCBI,16S: NR_024696] (T)		*Bacillus vallismortis*	100%	[KF254573,74]
	2,12	[Greengenes: FJ907189]		*Bacillus mojavensis*	100%	[KF254575,79,81]
	3	[NCBI,NC: JQ917920]		*Bacillus atrophaeus*	100%	[KF254576]
	3	[NCBI,NC: AB681416] (T)		*Bacillus sonorensis*	99%	[KF254578]
	2	[NCBI,NC: JX290089]	insect to human pathogens	*Bacillus cereus* group	100%	[KF254677-78]
	6	[NCBI,16S: NR_042072] (T)	soil; sediment; sspoiled coconut; vermicompost; gut of estuarine fish	*Lysinibacillus fusiformis*	99%	[KF254734]
	2	[EzTaxon-e: FJ386524]	salt lake; a moderately halophilic bacterium	*Terribacillus aidingensis*	100%	[KF254730-31]
XII. Incertae Sedis	2	[Greengenes: AJ846291]	psychrophilie and alkaliphile; water; *Anopheles stephensi*	*Exiguobacterium indicum*	99.86%	[KF254737]
Paenibacillaceae	3	[Greengenes: AY359885]	blood culture, *Phlebotomus papatasi*; *Apis melifera*	*Paenibacillus dendritiformis*	98.34%	[KF254756]
	12	[EzTaxon-e: EU014873] (T)	sugar cane	*Saccharibacillus sacchari*	97%	[KF254732]
Planococcaceae	9	[NCBI,NC: HM854242]	endophyte of *Populus euphractica*	*Planomicrobium okeanokoites*	99%	[KF254583]
	2	[leBIBI: EU036220] (T)	glacier	*Planomicrobium glaciei*	99.86%	[KF254584]
	10	[NCBI,NC: JX290556]	soy sauce	*Sporosarcina luteola*	99%	[KF254736]
Staphylococcaceae	3	[Greengenes: DQ361017]	commensal of the skin of humans and animals; meat fermentator, animal opportunistic infections*, Musca domestica*, *Calliphora spp*	*Staphylococcus xylosus*	100%	[JX661717]
	1,2,10,12	[NCBI,NC: CP003194]	skin and respiratory tract of human, *Phlebotomus argentipes*	*Staphylococcus aureus*	100%	[KF254613,20, 26,34,38-57]
	6	[Greengenes: HM113469]	human urinary tract infections, *Phlebotomus papatasi*, *Phlebotomus argentipes*, *Musca domestica*	*Staphylococcus saprophyticus*	100%	[KF254614-16,22]
	2,4,8	[DDBJ: L37605]	animal and human skin/mucous; nosocomial pathogen associated with infections of implanted medical device	*Staphylococcus epidermidis*	100%	[KF254617,19,23,25,30,32,35,37]
	3	[EzTaxon-e: AJ421446]	human; farm animals; pets; wild animals; food products of animal origin	*Staphylococcus sciuri*	100%	[KF254618,28]
	2,6,8	[EzTaxon-e: AF004220]	cheese; sausages; skin of healthy wild animals; human clinical specimens; Dominican amber	*Staphylococcus succinus*	99.93%	[KF254621, 24, 29]
	2	[Greengenes: L37601]	commensal on human and animal skin; human pathogens in immunocompromised patients	*Staphylococcus hominis*	99.86%	[KF254627]
	2	[EzTaxon-e: L37603]	commensal on human and animal skin; human pathogens in immunocompromised patients	*Staphylococcus warneri*	100%	[KF254631]
	2	[EzTaxon-e: EU888120]	viscera of common voles	*Staphylococcus microti*	100%	[KF254633]
Enterococcaceae	4,6,7,8,9,10,11	[Greengenes: FJ378679]	gastrointestinal tract colonizers with lifestyles ranging from intestinal symbiont to environmental persister to multidrug-resistant nosocomial pathogen	*Enterococcus faecium*	100%	[KF254533,34,36-41,43-50,56,57,60,61, JX661714]
	1,3	[Greengenes: FJ607291]		*Enterococcus faecalis*	99.86%	[KF254542,51,54,55]
	2,3	[Greengenes: FJ915740]		*Enterococcus gallinarum*	99.93%	[KF254535,52,53,59]
	4	[NCBI,NC: AB680105]		*Enterococcus casseliflavus*	100%	[KF254558]
Brucellaceae	10	[EMBLE: FJ950543]	soil; oxytetracycline production wastewater	*Ochrobactrum grignonense*	99%	[KF254738]
Alcaligenaceae	2	[NCBI,NC: JN575638]	environment; pet birds; human opportunistic infections	*Alcaligenes faecalis*	99%	[KF254754]
Enterobacteriaceae	3	[EzTaxon-e: AJ233408]	soil, water; sewages; mammals; birds; reptiles; amphibians; biodegradator of tannic acid	*Citrobacter freundii*	99.93%	[KF254749]
	2	[RDP: AF025369]	human clinical samples and food	*Citrobacter murliniae*	99.13%	[KF254750-52]
	2,3	[Greengenes: FJ463820]	gut of humans and other warm-blooded animals	*Escherichia coli*	99.72%	[KF254747, 48]
	2,3,5	[Greengenes: HM362787]	terrestrial and aquatic environments; as normal flora of plants; insects; humans	*Enterobacter ludwigii*	99.02%	[KF254586, 590,593,596,600,753]
	3,6,10,11	[Greengenes: HM058581]		*Enterobacter hormaechei*	99.79%	[KF254587,88,94,97, JX661718]
	3,6	[Greengenes: GU549440]		*Enterobacter cloacae*	100%	[KF254589,91,95]
	3	[Greengenes: AY335554]		*Enterobacter aerogenes*	100%	[KF254592,601]
	3	[NCBI,NC: JQ795788]		*Enterobacter asburiae*	100%	[KF254598]
	6	[EzTaxon-e: Z96078]		*Enterobacter cancerogenus*	99.15%	[KF254599]
	1	[NCBI,NC: Z96079] (T)	soil; candidate for 2,3-butanediol production; pathogen of maize and mulberry; endophyte of *Populus euphratica*; indigenous bacterium of zebrafish gut; PGPR of *Tripterygium wilfordii’s*; rhizosphere of symptomatic and asymptomatic plants of maize for *Fusarium verticillioides*; moderately halophilic bacterium from marine sediment; rhizosphere of rice and soybean; hutti gold mine water	*E. cloacae dissolvens*	99.93%	[KF254602]
	3,4,5	[NCBI,16S: NR_041749] (T)	a zoonose agent; nosocomial infections	*Klebsiella oxytoca*	100%	[KF254664-66]
	2	[EMBLE: AP012032]	PGRP of potato and pepper; epiphyte and entophyte of plants, is capable of infecting humans; occurs in diverse ecological niches	*Pantoea ananatis*	100%	[KF254658-61]
	2	[NCBI,NC: JF799896]	part of the normal flora of the human gastrointestinal tract; when entering, causes urinary tract infections and the formation of stones; interkingdom swarming signals attract blow flies; melanoidin and heavy metals degrading bacterium; urine; fishmeal sample	*Proteus mirabilis*	100%	[KF254662,63]
Moraxellaceae	2,3	[DDBJ: JN644621]	soil, water; wastewater; as nosocomial pathogen;			
Effluent of treatment plant laden with hydrocarbons; PGPR; Mushroom compost	*Acinetobacter calcoaceticus*	100%	[KF254603-4,607,609,611]			
	2	[EzTaxon-e: ACQB01000091]		*Acinetobacter baumannii*	100%	[KF254608,10]
	3	[NCBI,16S: NR_044454] (T)	soil; raw milk; wastewater; nosocomial pathogen; midgut of culex quinquefasciatus, mealybug; of ficus deltoidea Jack	*Acinetobacter soli*	100%	[KF254605,606,612]
Pseudomonadaceae	1,2	[EzTaxon-e: Z76651] (T)	human clinical speciments; rhizosphere of *Nicotiana glauca*; endophyte of black pepper; soil; coastline	*Pseudomonas aeruginosa*	99.93%	[KF254520-25,27-32, JX661716]
	8	[EzTaxon-e: GQ161991] (T)	soil	*Pseudomonas bauzanensis*	99.72%	[KF254526]
Xanthomonadaceae	2,9	[EMBLE: GQ360071]	rhizosphere of common bean, *Nicotiana glauca*; human sputum	*Stenotrophomonas maltophilia*	99.72%	[KF254498,502,506,510,512,514-519]
	1,2,9	[EzTaxon-e: AB021404]	PGPR*;* endophtic bacteria of invasive and stress resistant plants; chironomid egg masses; rhizosphere of rice, tobacco, maize; cow manure; endosymbionts of cotton leaf hopper and aphids; shrimp *Penaeus merguiensis*	*Pseudomonas geniculata*	100%	[KF254489-97,499-501,503-505,507-509,511,513, JX661715]

AN: Accession Number, *Isolation source: 1) Male sandfly midgut, 2) Female sandfly midgut, 3) Rodent skin, 4) Rodent feces, 5) Rodent viscera, 6) Imported diet plants, 7) Imported wool fiber, 8) Nest soil, 9) Epiphyte of Hyloxylon, 10) Endophyte of Hyloxylon, 11) Epiphyte of Salsola and 12) Endophyte of Salsola.

**Data bases: Greengenes, EzTaxon-e, NCBI (16 s rRNA sequences), NCBI (Nucleotide collection), EMBLE, DDBJ, leBIBI, RDP and Blast2Tree.

### Sand fly gut bacteria

We isolated 170 bacterial isolates from the guts of *P. papatasi* which included 40 distinct species (Table 3). The bacteria comprised pathogens, e.g. *Acinetobacter calcoaceticus-baumannii* complex*, Escherichia coli, Enterobacter cloacae* complex*, Pseudomonas aeruginosa, Staphylococcus aureus,* and *Stenotrophomonas* spp, whereas others were non-pathogenic or rare–pathogenic organisms. Members of families Microbacteriaceae, Staphylococcaceae and Xanthomonadaceae were the most prevalent bacteria in the sand flies guts. Also *Microbacterium paraoxydans* was generally found in the greatest abundance.

**Table 3:** **Frequency of the bacteria isolated from** ***P. papatasi*** **guts based on their habitats**

	**Rodent colony**		**Cattle farm**		**Human dwelling**		**No.**
	**Female**	**Male**	**Female**	**Male**	**Female**	**Male**	
*Acinetobacter calcoaceticus*	1	---	---	---	---	---	1
*Alcaligenes faecalis*	1	---	---	---	---	---	1
*Microbacterium sediminis*	1	---	---	---	---	---	1
*Staphylococcus warneri*	1	---	---	---	---	---	1
*Staphylococcus succinus*	1	---	---	---	---	---	1
*Microbacterium paraoxydans*	1	---	---	---	45	---	46
*Pseudomonas geniculata*	8	2	9	1	---	---	20
*Staphylococcus aureus*	13	2	6	---	---	1	22
*Staphylococcus epidermidis*	5	---	---	---	1	---	6
*Stenotrophomonas maltophilia*	5	---	5	---	---	---	10
*Exiguobacterium indicum*	1	---	---	---	---	---	1
*Proteus mirabilis*	2	---	---	---	---	---	2
*Bacillus flexus*	1	---	---	---	---	1	2
*Bacillus mojavensis*	1	---	1	---	---	---	2
*Enterococcus gallinarum*	2	---	---	---	---	---	2
*Enterobacter ludwigii*	3	---	1	---	---	---	4
*Pseudomonas aeruginosa*	1	---	11	1	---	---	13
*Bacillus licheniformis*	1	---	---	---	---	---	1
*Bacillus cereus group*	1	---	1	---	---	---	2
*Bacillus amyloliquefaciens*	1	---	1	---	---	---	2
*Bacillus subtilis subsp spizizenii*	1	---	---	---	---	---	1
*Streptomyces roseofulvus*	1	---	---	---	---	---	1
*[Brevibacterium] frigoritolerans*	1	---	---	---	---	---	1
*Citrobacter murliniae*	1	---	2	---	---	---	3
*Pantoea ananatis*	---	---	4	---	---	---	4
*Bacillus vallismortis*	---	---	1	1	---	---	2
*Escherichia coli*	---	---	1	---	---	---	1
*Bacillus endophyticus*	---	---	1	---	---	---	1
*Terribacillus aidingensis*	---	---	1	---	1	---	2
*Planomicrobium glaciei*	---	---	1	---	---	---	1
*Staphylococcus hominis*	---	---	1	---	---	---	1
*Acinetobacter baumannii*	---	---	2	---	---	---	2
*Microbacterium imperiale*	---	---	---	---	1	---	1
*Staphylococcus microti*	---	---	---	---	1	---	1
*Bacillus firmus*	---	---	---	---	1	---	1
*Bacillus circulans*	---	---	---	---	2	---	2
*Bacillus pumilus*	---	---	---	---	2	---	2
*Bacillus altitudinis*	---	---	---	1	---	---	1
*Enterobacter cloacae dissolvens*	---	---	---	---	---	1	1
*Enterococcus faecalis*	---	---	---	---	---	1	1
Total	55	4	49	4	54	4	170

Results showed bacterial diversity among sand fly gut in the three regions studied where we found 24, 18, and 12 bacterial species from rodent colony, cattle farm and human dwellings, respectively. *Staphylococcus aureus* was the only common bacterium in guts of sand flies of three regions. Comparison of the bacteria isolated from guts of the three locations indicated that species of *Microbacterium, Pseudomonas*, and *Staphylococcus* genera were dominant in human dwellings, cattle farms, and rodent colonies, respectively.

### Bacteria circulation in micro- and macroclimate levels

In this study we simultaneously identified microflora of all wild components of ZCL cycle presented in and around a single rodent colony (microclimate) located between the Abbas Abad village and the Agha-ali Abbas shrine. 83 aerobic bacterial strains were isolated from the biotic and abiotic parts of the colony where 59 isolates were from the sand fly guts and 24 isolates were from other compartments (Table 4, data shown in parentheses). Both *Pseudomonas geniculata* and *Staphylococcus aureus* were present in male and female guts. These two bacteria were also found on the surface of *Haloxylon* plants and internal tissues of *Salsola* plants. *Staphylococcus epidermidis* were found in the female midguts, the rodent faeces and the nest soils. *Bacillus mojavensis* was found in the female sand fly guts and internal tissues of *Salsola* plants. Different strains of *Bacillus subtilis* were found in the female sand fly guts as well as in the nest plant pieces, and as epiphytes on both *Salsola* and *Haloxylon* plants. Details of spatial distribution of the bacteria isolated from different partners of the rodent colony are shown in Table 4 (data shown in parentheses).

**Table 4:** **Details of the isolated bacteria arranged by their sources**

**Isolation source**	**MM**	**FM**	**RS**	**RF**	**RV**	**NP**	**NF**	**NSo**	**EH**	**NH**	**ES**	**NS**	**No.**
**Bacteria**													
*Arthrobacter bergere*								(1)					1
*Arthrobacter arilaitensis*						1							1
*Arthrobacter nicotianae*			1										1
*Microbacterium sediminis*		(1)											1
*Microbacterium imperiale*		1											1
*Microbacterium paraoxydans*		48(1)											49
*Streptomyces roseofulvus*		(1)											1
*Myroides odoratus*			4	(1)									5
*Wautersiella falsenii*			1										1
*Brevibacterium frigoritolerans*		(1)											1
*Bacillus endophyticus*		1											1
*Bacillus flexus*	1	(1)											2
*Bacillus firmus*		1											1
*Bacillus circulans*		2											2
*Bacillus pumilus*		2				1			1				4
*Bacillus altitudinis*	1												1
*Bacillus amyloliquefaciens*		1(1)	1					1					4
*Bacillus subtilis subsp spizizenii*		1(1)				(1)	1		(1)				5
*Bacillus subtilis subsp subtilis*											(1)		1
*Bacillus subtilis*								1	2		1		4
*Bacillus licheniformis*		(1)											1
*Bacillus vallismortis*	1	1											2
*Bacillus mojavensis*		1(1)										(1)	3
*Bacillus atrophaeus*			1										1
*Bacillus sonorensis*			1										1
*Bacillus cereus group*		1(1)											2
*Lysinibacillus fusiformis*						(1)							1
*Terribacillus aidingensis*		2											2
*Exiguobacterium indicum*		(1)											1
*Paenibacillus dendritiformis*			1										1
*Saccharibacillus sacchari*												1	1
*Planomicrobium okeanokoites*									1				1
*Planomicrobium glaciei*		1											1
*Sporosarcina luteola*										1			1
*Staphylococcus xylosus*			(1)										1
*Staphylococcus aureus*	1(2)	6(13)								1		(1)	24
*Staphylococcus saprophyticus*						(4)							4
*Staphylococcus epidermidis*		1(5)		(1)				(1)					8
*Staphylococcus sciuri*			(2)										2
*Staphylococcus succinus*		(1)				1		1					3
*Staphylococcus hominis*		1											1
*Staphylococcus warneri*		(1)											1
*Staphylococcus microti*		1											1
*Enterococcus faecium*				5		3	2	6(1)	2	1	1		21
*Enterococcus faecalis*	1		3										4
*Enterococcus gallinarum*		(2)	2										4
*Enterococcus casseliflavus*				1									1
*Ochrobactrum grignonense*										1			1
*Alcaligenes faecalis*		(1)											1
*Citrobacter freundii*			1										1
*Citrobacter murliniae*		2(1)											3
*Escherichia coli*		1	1										2
*Enterobacter ludwigii*		1(3)	1		1								6
*Enterobacter hormaechei*			2			1				(1)	(1)		5
*Enterobacter cloacae*			2			(1)							3
*Enterobacter cloacae dissolvens*	1												1
*Enterobacter aerogenes*			2										2
*Enterobacter asburiae*			1										1
*Enterobacter cancerogenus*						1							1
*Klebsiella oxytoca*			1	(1)	1								3
*Pantoea ananatis*		4											4
*Proteus mirabilis*		(2)											2
*Acinetobacter calcoaceticus*		(1)	4										5
*Acinetobacter baumannii*		2											2
*Acinetobacter soli*			2(1)										3
*Pseudomonas aeruginosa*	1	11(1)											13
*Pseudomonas bauzanensis*								1					1
*Stenotrophomonas maltophilia*		5(5)							1				11
*Pseudomonas geniculata*	1(2)	9(8)							(1)				21
Total	12	162	36	9	2	15	3	13	9	5	4	3	273

Numbers in the parenthesis indicate presence of all four partners (vector, reservoir, nest materials and food sources) of ZCL in the micro-climate.

*MM* Male sandfly midgut, *FM* Female sandfly midgut, *RS* Rodent skin exposed area, *RF* Rodent feces, *RV* Rodent viscera, *NP* Nest imported diet plants, *NF* Nest imported wool fiber, *NSo* Nest soil, *EH* Epiphyte of *Hyloxylon*, *NH* Endophyte of *Hyloxylon*, *ES* Epiphyte of *Salsola* and *NS* Endophyte of *Salsola*.

At the macroclimate level, analyses were performed on all isolates found in the whole area of study and the bacteria were assembled in Table 4 according to their isolation origins. Comparative analysis revealed that in 16 cases the bacterial isolates were present in both micro- and macroclimates (Table 4).

**Figure 4:**
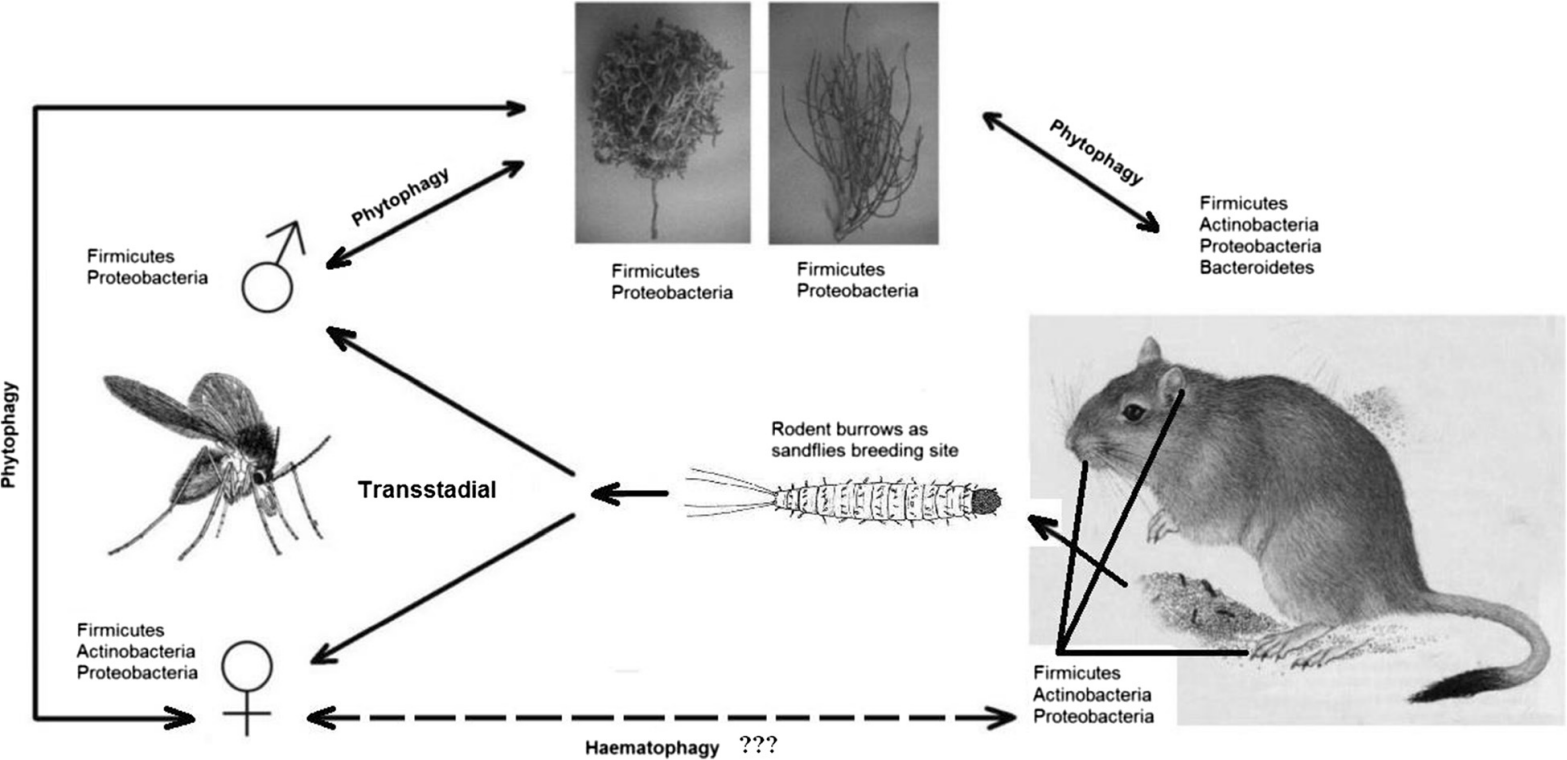
**Natural circulations of bacteria among the partners of ZCL focus in Isfahan, Iran.** Operators *P. papatasi*, *R. opimus* and plant food sources (*Hyloxylon* and *Salsola*) indicated by graphics. One or two way arrows show how the bacteria are acquired and circulated. Continuous and non-continuous lines represent proved and doubtful transmission routes respectively. Bacteria phyla engrave close to the isolated source.

Analyses of bacteria at the macroclimate level simulated bacterial circulation pattern among four ZCL operators. In this way selection of suitable candidates and their possible application routes were disclosed. This model demonestrates how bacteria are circulated among other ZCL partners by sand flies (Figure 4).

### Sand fly oviposition inducing bacteria

In this study we found 12 isolates of soil bacteria comprising eight bacilli and four coagulase-negative staphylococci that are known to be involved in the inter-kingdom communication of eukaryotic sand flies/plants and prokaryotic bacteria. Details of the bacteria that may be considered to enhance the oviposition response of gravid *P. papatasi* females are shown in Table 5.

**Table 5:** **Details of the oviposition inducer bacteria were found in this study** [84]

**Isolated bacteria**	**Isolation source (n)**	**No.**
*Bacillus pumilus*	Epiphyte of Hyloxylon (1)	4
	Imported nest plants (2)	
	Sandfly midgut (1)	
*Bacillus cereus* group	Sandfly midgut	2
*Bacillus firmus*	Sandfly midgut	1
*Staphylococcus saprophyticus*	Imported nest plants	4
*Bacillus licheniformis*	Sandfly midgut	1
Total		12

### Potential routes of bacterial acquisition by sand flies

Adult *P. papatasi* midgut bacteria might be acquired from three general routes: adult sugar feeding on the plant saps, adult blood feeding on the animal host and larval feeding in their breeding places that would be transmitted transstadially from larvae to adult. There were five common bacteria present in both male and female guts: *Pseudomonas geniculata* (male = 3, female = 17), *P. aeruginosa* (1, 12), *Bacillus vallismortis* (1, 1), *B. flexus* (1, 1), and *Staphylococcus aureus* (3, 19). The habits of feeding on plant saps by both adult female and male sand flies and of feeding on organic materials by larvae can explain the presence of those common bacteria in their digestive tracts. Comparison of the bacterial content of the nest soil, rodent feces, the imported plant diets, and the wool fibres specimens with the bacteria found in the gut of adult sand flies verified the possibility of a transstadial transmission mode of 7 isolates during transition from breeding places materials (immature midgut) to adult midgut (Table 6).

**Table 6:** **Possible routes of bacteria to enter sandfly gut**

**Isolation source**		**Isolated bacteria**														
		***AC***	***EL***	***EC***	***BA***	***EG***	***ECo***	***BP***	***BM***	***BS***	***SS***	***SE***	***SA***	***PGe***	***SM***	***PGl***
Female sand fly gut		1	4	1	2	2	1	2	2	2	1	6	19	17	10	1
Exposed area of the rodent skin		4	1	---	1	2	1	---	---	---	---	---	---	---	---	---
Epi/Endophytes of *Hyloxylon* and *Salsola*		---	---	---	---	---	---	1	1	5	---	---	2	1	1	1
Larval breeding places	Nest soil	---	---	---	1	---	---	---	---	1	1	1	---	---	---	---
	Rodent feces	---	1	---	---	---	---	---	---	---	---	1	---	---	---	---
	Imported diet plants	---	---	1	---	---	---	1	---	1	1	---	---	---	---	---
	Imported wool fibers	---	---	---	---	---	---	---	---	1	---	---	---	---	---	---

The first row represents the bacteria found in the female sand fly guts where other rows indicate their correspondence sources. *AC: Acintobacter calcoaceticus, EL: Enterobacter ludwigii, EC: Enterobacter cloacae, BA: Bacillus amyloliquefaciens, EG: Enterococcus gallinarum, ECo: Escherichia coli, BP: Bacillus pumilus, BM: Bacillus mojavensis, BS: Bacillus subtilis, SS: Staphylococcus succinus, SE: Staphylococcus epidermidis, SA: Staphylococcus aureus, PGe: Pseudomonas geniculata, SM: Stenotrophomonas maltophilia* and *PGl: Planomicrobium glaciei.*

Concerning the sand fly midgut bacteria and the routes that allow them to enter during insect feeding, it was revealed that 5 species of *Acinetobacter calcoaceticus*, *Enterobacter ludwigii*, *Bacillus amyloliquefaciens*, *Enterococcus gallinarum* and *Escherichia coli* might be acquired when blood feeding on the reservoir host and 7 species of *B. pumilus*, *B. mojavensis*, *B. subtilis*, *S. aureus*, *Stenotrophomonas maltophilia*, *Pseudomonas geniculata* and *Planomicrobium* spp. might be obtained when feeding on the plant saps (Table 6).

The bacterium *Microbacterium paraoxydans* was isolated from different physiological states (unfed, fed, semi-gravid and gravid) of female sand flies caught from an uninhabited home in the Parvaneh village of Habib-abad district, indicating that the isolate bacterium could tolerate blood digestion and gonotrophic processes (Table 7). Bacterial flora comparisons before and after blood feeding showed that some bacterial strains remain after blood digestion but in general there were variations in bacterial compositions (Table 8).

**Table 7:** Bacteria isolated from the sand fly guts at different abdominal stages*

**Source**			**Isolated bacteria**	**No.**
Female	Individually dissected guts	Unfed	*Microbacterium paraoxydans*	8
			*Microbacterium imperiale*	1
		Blood fed (naturally or laboratory)	*Microbacterium paraoxydans*	34
			*Staphylococcus microti*	1
			*Bacillus firmus*	1
			*Terribacillus aidingensis*	1
			*Bacillus circulans*	1
			*Staphylococcus epidermidis*	1
		Semi-gravid	*Bacillus pumilus*	1
		Gravid	*Microbacterium paraoxydans*	1
	Multiple dissected guts	Unfed	*Microbacterium paraoxydans*	2
			*Bacillus circulans*	1
		Insect whole body	*Bacillus pumilus*	1
Male	Bundles of quintuple dissected guts		*Bacillus flexus*	1
			*Staphylococcus aureus*	1
			*Enterobacter cloacae dissolvens*	1
			*Enterococcus faecalis*	1
Total				58

*Sand flies captured in an uninhabited home in the Parvaneh village of Habib-abad district.

**Table 8:** **The sandfly gut bacteria before and after blood feeding on** ***R. opimus***

**Collection site**	**Immediately after collection**	**No**	**24 hrs after blood feeding**	**No**
**Rodent colony**	***Proteus mirabilis***	**1**	***Proteus mirabilis***	**1**
	***Pseudomonas geniculata***	**5**	***Pseudomonas geniculata***	**3**
	***Stenotrophomonas maltophilia***	**4**	***Stenotrophomonas maltophilia***	**1**
	***Staphylococcus aureus***	**9**	***Staphylococcus aureus***	**4**
	***Staphylococcus epidermidis***	**4**	***Staphylococcus epidermidis***	**1**
	***Enterobacter ludwigii***	**1**	***Enterobacter ludwigii***	**2**
	***Bacillus subtilis subsp spizizenii***	**1**	***Bacillus subtilis subsp spizizenii***	**1**
	*Acinetobacter calcoaceticus*	1	*Bacillus cereus group*	1
	*Alcaligenes faecalis*	1	*Bacillus licheniformis*	1
	*Microbacterium sediminis*	1	*Streptomyces roseofulvus*	1
	*Staphylococcus warneri*	1	*[Brevibacterium] frigoritolerans*	1
	*Bacillus mojavensis*	1	*Citrobacter murliniae*	1
	*Staphylococcus succinus*	1	*Bacillus amyloliquefaciens*	1
	*Microbacterium paraoxydans*	1	*---*	*---*
	*Exiguobacterium indicum*	1	*---*	---
	*Enterococcus gallinarum*	2	*---*	---
	*Pseudomonas aeruginosa*	1	*---*	---
	*Bacillus flexus*	1	*---*	---
	**Total**	**37**	**Total**	**19**
**Cattle farm**	***Pantoea ananatis***	**3**	***Pantoea ananatis***	**1**
	***Pseudomonas geniculata***	**3**	***Pseudomonas geniculata***	**6**
	***Stenotrophomonas maltophilia***	**2**	***Stenotrophomonas maltophilia***	**3**
	***Citrobacter murliniae***	**1**	***Citrobacter murliniae***	**1**
	***Staphylococcus aureus***	**5**	***Staphylococcus aureus***	**1**
	***Pseudomonas aeruginosa***	**1**	***Pseudomonas aeruginosa***	**10**
	*Bacillus vallismortis*	1	*Bacillus amyloliquefaciens*	1
	*Escherichia coli*	1	*---*	---
	*Bacillus endophyticus*	1	*---*	---
	*Enterobacter ludwigii*	1	*---*	---
	*Terribacillus aidingensis*	1	*---*	---
	*Planomicrobium glaciei*	1	*---*	---
	*Bacillus mojavensis*	1	*---*	---
	*Staphylococcus hominis*	1	*---*	---
	*Acinetobacter baumannii*	2	*---*	---
	*Bacillus cereus group*	1	*---*	---
	**Total**	**26**	**Total**	**23**

Bold and non-bold spp indicate identical and non-identical isolates respectively. The collection sites were in the Abbas-abad village of Bad-rood city.

### Possible bacterial acquirement of sand fly gut via blood feeding

A group of sand flies were allowed to feed on rodents and 24 hours after blood ingestion their gut contents were examined for the presence of the bacteria originally isolated on the rodent skin. None of the rodent skin bacteria was found in the female gut. Details of the bacteria that were isolated from the control specimens are shown in Table 9. Comparison of skin surface bacteria of field and lab rodents showed no resemblance except for the presence of *Enterococcus faecalis*.

**Table 9:** **Contamination controls used in this study**

**Contamination sources**			**Isolated bacteria**	**No.**
Sandflies		The rest of the body (other than gut lumen)	*Microbacterium paraoxydans*	3
*R. opimus*	Wild	Alimentary canal of a dissected *R. opimus*	*Enterobacter ludwigii*	1
			*Klebsiella oxytoca*	1
		Skin surface of *R. opimus* used for blood feeding of entraped sandflies from a rodent colony in the Abbas-abad village of Badrood city	*Enterococcus faecalis*	1
		Skin surface of *R. opimus* used for blood feeding of entraped sandflies from a semi-desolated cattle farm in the Abbas-abad village in the Bad-rood city	*Escherichia coli*	1
			*Enterococcus faecalis*	1
		Skin surface of *R. opimus* used for blood feeding of entraped sandflies from an uninhabited home in the Parvaneh village of Habib-abad district	*Enterobacter ludwigii*	1
	Animal house	Skin surface of *R. opimus* reared in the Isfahan Health Research Station animal house.	*Acinetobacter calcoaceticus*	1
			*Bacillus atrophaeus*	1
			*Bacillus sonorensis*	1
			*Bacillus amyloliquefaciens*	1
			*Enterococcus gallinarum*	2
			*Paenibacillus dendritiformis*	1
			*Enterococcus faecalis*	1
Environmental		Medium contamination due to lab condition	*Staphylococcus epidermidis*	1
Total				18

## Discussion

In this study we investigated the microbiology of the biotic and abiotic compartments of a natural ZCL cycle, including the gut of the sand fly vector *P. papatasi*, skin and internal organs of the animal reservoir *R. opimus*, natural plants normally used as food for both vector and reservoir, soils and other materials present in rodent nests and sand fly larval breeding places. Data analyses showed that bacteria flora encompass a wide range of aerobic to facultative anaerobic, harmless commensals to important pathogens, inter-cellular to intra-cellular, environmental to nosocomial pathogens, skin surface to gut lumen bacteria, endophytes to epiphytes, extremophiles to mesophiles or neutrophiles, land to air and ocean habitat, animal and human probiotics to plant growth-promoting rhizobacteria (PGPR) (Table 2).

This study shows an association between the microbiota of the sand fly gut and the places they live in; a number of the isolates identified in the sand fly guts were also present in the *R. opimus* nest materials/sand fly larval breeding places and the plants which were used by vectors as sugar sources or by rodents for food and water. The association between the microbiota of the sand fly gut and larval breeding sites supports transstadial transmission of some bacteria; however, some authors argue against the transstadial route and believe that full gut turnover occurs during pupation [73]. Environmental acquisition of sand fly gut bacteria has been reported by other investigators [57,74]. These studies suggest that the sand fly gut microbiota is a reflection of both the environment in which the sand fly larvae reside and the food sources of larvae and adults.

Comparison of bacterial diversity in the sand fly guts from three regions revealed that the microflora were largely environmental; the predominant bacteria were species of *Microbacterium, Pseudomonas*, and *Staphylococcus* in the human dwellings, cattle farm, and rodent colony, respectively. This diversity may be partly due to the kind and accessibility of sand fly hosts. The available hosts for sand flies of the human dwellings (Parvaneh village) were pigeons, sheep, and or humans, whereas available hosts for sand flies trapped from rodent’s colony and the cattle farm were rodent and cow, respectively. The effect of blood meal components on the growth and persistence of some microbes has been proven [75,76]. Other factors such as intestine-specific structure, pH, redox, digestive enzymes, and food sources are determinants of microbial colonization in insect guts [34,77,78]. It was noted that in insects with various diets, microbial growth kinetics are dissimilar and different types of bacteria are present in their guts. Dillon et al. [77] showed that the number of bacteria present in the gut of *P. papatasi* changes during the lifetime of a female. Volf et al. [29] showed that the highest bacterial counts occurred two days after blood ingestion. The protein rich bolus of the blood presumably caused rapid growth of midgut bacteria and when digestion is completed (on day 4–5) most bacteria were defecated with blood remains. Seven days after blood feeding the bacterial count returned to the pre-feeding level. It is suggested that blood digestion, the development of *Leishmania* parasites in the competent vector and bacterial population fluctuations are closely related to each other. Sant’Anna et al. [79] implied colonisation resistance in the *Lutzomyia longipalpis* and investigated the balance of microbiota and *Leishmania* in the insect gut. They found a reduction in the number of flies harboring a *Leishmania* population that had been pre-fed with *Pseudozyma* sp. and *Asaia* sp. or *Ochrobactrum intermedium*. Also they discovered that *L. mexicana* protects *Lu. longipalpis* from *Serratia marcescens* infection. They concluded that *Leishmania*-vector association might benefit for both the sand fly and parasite [79].

In the current study we found that Microbacteriaceae were the most frequently isolated bacteria (27%) in the *P. papatasi* digestive tract, which is in agreement with the previous study of sand fly gut microbiology of *P. papatasi* and *P. duboscqi* [28]. Also we observed presence of *Microbacterium* sp in different physiologic states of *P. papatasi* indicating resistance to trypsins and chymotrypsin enzymes during blood digestion (Table 8). These bacteria have already been found in the adult guts of *P. duboscqi* and *P. papatasi* [28]. Also presence of *Microbacterium* in the 2nd and 4th instar larvae, pupae, male and female adults of *P. duboscqi* suggested transstadial transmission. *Microbacterium* species was also reported in the guts of field trapped *P. argentipes* from India and of *Ixodes ricinus* [57,80]. Interestingly, various strains of *Microbacterium* isolated from *Musca domestica* guts have been shown to support its larval development [81]. It is demonstrated that gut microbiota also influence the sand fly immune systems. For example, the concentration of regulating gut-microbe homeostasis such as reactive oxygen species (ROS) will change in *Lu. longipalpis* midguts in response to *Leishmania* parasite or to insect pathogen *Serratia marcescens* [82]. Also it is shown that the rate of defensin expression in *Lu. longipalpis* upon bacterial and *Leishmania* infection will vary based on the bacterial species and the routes of *Leishmania* infection [83]. Further studies warrant showing the effect of gut microbiota on the immune system of *P. papatasi* the most important vector of ZCL in the Old World.

It is shown that oviposition by gravid *P. papatasi* is influenced by microbial flora of the environment. Radjame et al. [84] and Mukhopadhyay et al. [74] introduced bacteria to breeding sites, thereby attracting sand flies [74,84]. It was implied that the gravid sand flies found oviposition sites through attraction cues of four bacillus species *B. pumilus, B. cereus, B. firmus, B. licheniformis* and one Coagulase-negative staphylococcus, *Staphylococcus saprophyticus*. The current study revealed presence of these five oviposition-inducing bacteria in the study area (Table 5), however only two species of *B. pumilus* and *S. saprophyticus* were found in the great gerbil nest materials where sand flies lay eggs.

We found some bacteria in ZCL partners that can cause super infection in human lesions, which may hinder or prevent the healing process of ZCL. In a rural area of north Isfahan, the bacteria were isolated from 66.8% of ZCL and 64.7% of non-ZCL lesions. The most common species were *Staphylococcus aureus* and *S. epidermidis* followed by *Bacillus sp., Streptococcus pyogenes*, *Escherichia coli*, *Klebsiella sp., Proteus sp.*, *Enterobacter sp.* and *Pseudomonas aeroginosa* [85].

Results of this study established the presence of *Enterobacter cloacae* subsp. *dissolvens* and *Bacillus subtilis* in the digestive tract of sand flies as well as at the larval breeding sites in the great gerbil nest materials and plants that were part of rodent and insect diets. The association of these organisms with the sand flies makes them good candidates for use in a model of paratransgenesis. The two bacteria are commensal sand fly bacteria and could be transformed to deliver antileishmanial peptides within sand fly guts to prevent or to reduce *Leishmania* transmission. The transformed bacteria could be delivered easily on the plants and or sand fly larval breeding sites such as great gerbil nests, pigeon nests, and sheep and cattle sheds. The sand fly would encounter and be infected with the bacteria either at the larval stage in their breeding sites while feeding on organic materials or at the adult stage while taking sugar meals on plants. The phlebotomine sand flies, require sugar for survival and several different sources of sugar meals of insect origin (honeydew), and of plant origin, have been identified [42,86,87]. These sugar meals are often taken by feeding directly on tissues of plant organs including stems, leaves, and flowers [42,45,46]. Hurwitz et al. [88] showed transstadial passage of some bacteria in *P. argentipes* sand fly by introducing an engineered *Bacillus subtilis* expressing Green Fluorescent Protein (GFP) in sterilized larval chow and retrieved the glowing bacteria in the adult.

*Bacillus subtilis* harbour metabolites that exhibit activity against both the larval and pupal stages of mosquitoes [89] as well as plant pathogens [90]. It is one of the main bacteria used in industrial production of enzymes, antibiotics, fermented foods and vitamins [91,92]. *Enterobacter cloacae* is a member of the normal gut flora of many insects such as symbiotic or entomopathogenic and in the surface of vegetables. Several reports have been made with *E. cloacae* strains in the biological control of plant pathogens, such as *Phytium* spp., *Sclerotinia* sp*.*, *Rhizopus* sp., *Fusarium* spp. and many others [93]. Also it was shown that the bacterium significantly is able to block the *Plasmodium vivax* sporogonic development in *Anopheles albimanus* [93]. Currently Eappen et al. [94] showed that *E. cloacae* strongly induce expression of components of the mosquito immune response in the *An. stephensi* midgut.

*Enterobacter cloacae* have already been tested to deliver, express, and spread foreign genes in termite colonies [95]. Watanabe et al. [96] transformed *E. cloacae* with an ice nucleation gene to reduce the mulberry pyralid moth, *Glyphodes pyloalis*. Also Kuzina et al. [97] transformed *E. gergoviae* with the *Bacillus thuringiensis* toxin gene to control pink bollworm, (*Pectinophora gossypiella*) (Lepidoptera: Gelechiidae).

The present study and literature review revealed that *E. cloacae* subsp. *dissolvens* belong to the natural and stable flora of *P. papatasi*, and are amenable to isolation, culture and transformation with foreign genes. Although some species of *Enterobacter*, including *E. cloacae*, are potential human pathogens, these species have also been reported from rhizosphere of various crops exhibiting plant growth promoting abilities; just as *E. cloacae* subsp. *dissolvens* was found associated with rhizosphere of soybean under natural field conditions [98]. It was isolated from orchard soil samples in China, and is a potential industrial candidate for 2,3-Butanediol productions, which could produce more than 100 g/liter 2,3-BD from glucose [99]. *E. cloacae* subsp. *dissolvens* was also found in the endosphere of rice plants without causing apparent harm to the host plant [100,101]. Study of Rodrigues Neto et al. [102] showed low level of pathogenicity of the type strain of *E. cloacae dissolvens* on the onion.

Crucial requirements for the application of paratransgenesis in control of *Leishmania* is the ability to transform the isolate bacteria and to then test their potentially colonization rates in the sand flies and finally to assess their antileishmanial effect in laboratory and field conditions. There are some available antileishmanial molecules such as histatin 5, racemoside A, monoclonal antibodies, defensin A, and temporins [103-107]. We are currently started to transform the *E. cloacae* subsp. *dissolvens* isolate with defensin gene to test its efficacy against *L. major* in *in-vitro* condition. Our study is ongoing to enhance the expression and to assess the efficacy of the antileishmanial molecule in this genetically modified bacterium (unpublished data; data not shown). We have tested the transstadial situation of the candidate bacteria, and found that *E. cloacae* subsp. *dissolvens* was transstadial and transfer from larvae to adult stage that would permit delivery of transformed bacteria to the breeding sites of sand fly larvae.

## Conclusions

On the basis of our knowledge this study is the first culture-dependent molecular analysis of four important partners of ZCL cycle and could be used as an effective platform for future efforts to prevent leishmaniasis. This study revealed possible routes of acquisition of sand fly bacteria, which can provide proper application of transformed bacteria in the field. Also here we introduced two bacterial species of *Entrobacter cloacae* subsp. *dissolvens* and *Bacillus subtilis*, which are good candidates for paratransgenic control of the ZCL in the hyperendemic focus in central Iran.

## Abbreviations

ZCL: Zoonotic Cutaneous Leishmaniasis
NIHR-IHRS: National institute of health research, Isfahan health research station
BHI: Brain heart infusion
PGPR: Plant growth-promoting rhizobacteria
